# Identification of a Costimulatory Molecule Gene Signature to Predict Survival and Immunotherapy Response in Head and Neck Squamous Cell Carcinoma

**DOI:** 10.3389/fcell.2021.695533

**Published:** 2021-08-09

**Authors:** Ling Aye, Xiaole Song, Jingyi Yang, Li Hu, Xicai Sun, Jiaying Zhou, Quan Liu, Hongmeng Yu, Dehui Wang

**Affiliations:** ^1^Department of Otolaryngology, Eye & ENT Hospital, Fudan University, Shanghai, China; ^2^Research Units of New Technologies of Endoscopic Surgery in Skull Base Tumor, Chinese Academy of Medical Sciences, Shanghai, China

**Keywords:** immunotherapy, head and neck squamous cell carcinoma, prognosis, risk model, costimulatory molecule, tumor microenvironment

## Abstract

**Background:**

Head and neck squamous cell carcinoma (HNSCC) is one of the most common malignancies worldwide. Checkpoint blockade immunotherapy has made tremendous progress in the treatment of a variety of cancers in recent years. Costimulatory molecules constitute the foundation of cancer immunotherapies and are deemed to be promising targets for cancer treatment. This study attempted to evaluate the potential value of costimulatory molecule genes (CMGs) in HNSCC.

**Materials and Methods:**

Based on The Cancer Genome Atlas (TCGA) and Gene Expression Omnibus (GEO) dataset, we identified the prognostic value of CMGs in HNSCC. Subsequently, CMGs-based signature (CMS) to predict overall survival of HNSCC patients was established and validated. The differences of downstream pathways, clinical outcomes, immune cell infiltration, and predictive immunotherapy responses between different CMS subgroups were investigated via bioinformatic algorithms. We also explored the biological functions of TNFRSF12A, one risk factor of CMS, by *in vitro* experiments.

**Results:**

Among CMGs, 22 genes were related to prognosis based on clinical survival time in HNSCC. Nine prognosis-related CMGs were selected to establish CMS. CMS was an independent risk factor and could indicate the survival of HNSCC patients, the component of tumor-infiltrating lymphocytes, and the immunotherapy response rate. Functional enrichment analysis confirmed that CMS might involve immune-relevant processes. Additionally, TNFRSF12A was related to poor prognosis and enhanced malignant phenotype of HNSCC.

**Conclusion:**

Collectively, CMS could accurately indicate prognosis, evaluate the tumor immune microenvironment, and predict possible immunotherapy outcomes for HNSCC patients.

## Introduction

Head and neck cancer ranked as the seventh most common cancer overall (Bray et al., 2018), with an estimated 888,000 new cases globally in 2018. Head and neck squamous cell carcinoma (HNSCC) accounts for more than 90% of all head and neck tumors (Gupta et al., 2016). HNSCC is a disease with biological diversity and genomic heterogeneity, which originates from the squamous mucosa lining of the upper respiratory tract, including lip and mouth, nasal cavity, paranasal sinus, nasopharynx, oropharynx, larynx, and hypopharynx (Marur and Forastiere, 2016; Siegel et al., 2017).

More than 60% of HNSCC patients were diagnosed with cancerous lesions that were locally advanced or metastatic at the first visit. For patients suffering from locally advanced or metastatic disease, the 5-year overall survival (OS) rate is less than 50% (Lefebvre, 2005), with a local recurrence rate of 15–40% and a high risk of developing distant metastasis (Chow, 2020). Surgery remains the major treatment of choice for resectable HNSCC. For cases with unresectable tumors or in which surgery may lead to severe organ dysfunction, concurrent chemoradiotherapy is recommended as the standard treatment (Colevas et al., 2018). However, concurrent chemoradiotherapy may lead to serious long-term adverse events, including pharyngeal dysfunction, ototoxicity, neurotoxicity, and nephrotoxicity (Sklan and Collingridge, 2017).

As a new therapeutic pillar in various cancers, the PD-1 monoclonal antibody was approved by the FDA for the first-line treatment of unresectable recurrent or metastatic HNSCC in 2019 (Cohen et al., 2019). Since then, a new era of immunotherapy has begun. However, despite the advancements in cancer immunotherapies to date, there are still some unmet needs: the overall response rate was still suboptimal, and immunotherapeutic resistance also resulted in substantial barriers (Luke et al., 2017). In view of the *status quo* that current immunotherapy is largely based on affecting T cell function via costimulatory molecules (Waldman et al., 2020), a better understanding of the mechanisms and the roles of costimulatory molecules in these biological processes is needed to realize the full potential of this treatment approach.

Costimulatory molecules, which are composed of the B7-CD28 family (Zhang et al., 2018) and tumor necrosis factor (TNF) families (Ward-Kavanagh et al., 2016), constitute potential molecular targets for immunotherapeutic strategies (Shekarian et al., 2017). At present, 13 molecules are classified as B7-CD28 family members (Janakiram et al., 2015). The TNF family is comprised of the TNF ligand superfamily (TNFSF) and the TNF receptor superfamily (TNFRSF) with 48 molecules (Croft and Siegel, 2017). Nevertheless, the clinical significance and genome features of costimulatory molecule genes (CMGs) in HNSCC carcinogenesis were obscure yet.

In the current research, we systematically explored the expression pattern and prognostic significances of CMGs by bioinformatics analysis. The biological functions of TNFRSF12A were also investigated by *in vitro* experiments. Then the CMGs-based signature (CMS) was developed to predict prognosis, immunotherapy response, and immune cell infiltration for HNSCC patients.

## Materials and Methods

### Cell Culture and Transfection

The HNSCC cell line 6-10B and Tu 686 were purchased from the Cell Bank of the Chinese Academy of Sciences (Shanghai, China) and were grown under the recommended conditions. The vectors of plko-Puromycin-EGFP-shRNA-TNFRSF12A and plko-Puromycin-EGFP-NC were purchased from Ruoji Technology (Shanghai, China) and were transfected into 6-10B and Tu 686 cells. The shRNA1 sequences are GCAGGAGAGAGAAGTTCACCA(F) & CAAA TGCTGCAGTTCCTTAGT(R), and the shRNA2 sequences are AGGAGAGAGAAGTTCACCACC(F) & GGTGGTGAACTTC TCTCTCCT(R). These two target sequences were mixed with the same proportion. Subsequently, the cells with suitable fluorescence expression were screened with puromycin at a concentration of 4 μg/ml.

### Real-Time PCR

Total RNA was purified using Mini BEST Universal RNA extraction KIT (TaKaRa, Japan) and cDNA was synthesized using the Prime-Script RT Master Mix (TaKaRa, Japan) according to the manufacturer’s instructions. Real-time PCR was performed using SYBR Green Realtime PCR Master Mix (Yeasen, China). Samples from each experiment were analyzed in triplicate. The primer sequences used in this study were as follows:

GAPDH: GGACTCATGACCACAGTCCA(F) & CCAGTAG AGGCAGGGATGAT(R);
TNFRSF12A: TTTGGTCTGGAGACGATGC(F) & GGCTCT AGAATGGATGAATGAA(R);
CXCL2: CTCGCTGCGCCGGTTGCTGC(F) & GCTGCAGC GCAGCCCAGGCA(R);
CXCL11: GACGCTGTCTTTGCATAGGC(F) & GGATTTA GGCATCGTTGTCCTTT(R);
CCL19: CTGCTGGTTCTCTGGACTTCC(F) & AGGGATG GGTTTCTGGGTCA(R);
CXCL17: TGCTGCCACTAATGCTGATGT(F) & CTCAGG AACCAATCTTTGCACT(R);
CXCL3: CGCCCAAACCGAAGTCATAG(F) & GCTCCCCT TGTTCAGTATCTTTT(R).

### Enzyme-Linked Immunosorbent Assay (ELISA)

To detect the secreted CXCL2, CXCL11, and CCL19 levels, cell culture supernatants were harvested after 48 h and analyzed using CXCL2, CXCL11, and CCL19 Human enzyme-linked immunosorbent assay (ELISA) Kit (Yuanmin Biotechnology, Shanghai, China) according to the manufacturer’s protocols.

### Western Blot

Werstern Blot was performed according to previous research (Song et al., 2016). Specifically, the antibodies utilized in this research were listed as follows:

TNFRSF12A (1:1000, abs137500, Absin, Shanghai, China);
GAPDH (1: 1000, AF1186, Beyotime, Shanghai, China);
HRP-conjugated secondary antibodies:(1:1000, A0208, Beyotime, Shanghai, China).

### Cell Proliferation Assays and Migration Assays

Cell proliferation was detected by the cell counting kit-8 (CCK-8) and clone formation assays. In brief, cells were inoculated into 96-well plates (1000 cells/well). At each time point (1st, 2nd, 3rd, 4th, and 5th day), 10 μl of CCK-8 solution (Yeason, Shanghai, China) was added to the sextuplicate wells. The plates were incubated for 1.5 h and detected.

For clone formation assays, cells were seeded in a six-well plate (1000 cells/well) with the culture medium refreshed every 3 days for 2 weeks. Following the 2 weeks, the cells were washed with PBS, fixed, stained, and counted.

For migration assays, cells were incubated using 24-well transwell plates (8-μm pore size, Corning, NY, United States). Certain cells suspended in serum-free medium were plated in the upper chambers, and 0.6 ml of RPMI-1640 medium with 10% FBS was added to the lower chamber. After incubation for a suitable amount of time, the cells were fixed, stained, and counted under a microscope.

### mRNA Expression Datasets and Clinical Information

The expression profile of HNSCC and corresponding clinical information were downloaded from the Cancer Genomics Browser of University of California Santa Cruz (UCSC). This prognostic feature was further validated based on an independent data set (GSE65858) from the Gene Expression Omnibus (GEO) database (Wichmann et al., 2015). For the genes with several probes, mean expression values were recognized as the expression data. The clinical characteristics of these patients from multiple institutions are summarized in Table 1.

**TABLE 1:** Clinical characteristics of enrolled groups.

Characteristic	Level	TCGA cohort	GEO cohort
*n*		501	270
Futime	(Mean (SD))	909.87 (881.90)	883.03 (451.72)
Age	(Mean (SD))	61.08 (11.90)	60.12 (10.34)
Gender (%)	Female	134 (26.7)	47 (17.4)
	Male	367 (73.3)	223 (82.6)
Stage (%)	I	20 (4.0)	18 (6.7)
	II	96 (19.2)	37 (13.7)
	III	109 (21.8)	37 (13.7)
	IV	276 (55.1)	178 (65.9)
T (%)	T1	35 (7.0)	35 (13.0)
	T2	149 (29.7)	80 (29.6)
	T3	136 (27.1)	58 (21.5)
	T4	181 (36.1)	97 (35.9)
N (%)	N0	245 (48.9)	94 (34.8)
	N1	85 (17.0)	32 (11.9)
	N2	160 (31.9)	132 (48.9)
	N3	7 (1.4)	12 (4.4)
	NX	4 (0.8)	0 (0.0)
M (%)	NA	1 (0.2)	0 (0.0)
	M0	485 (96.8)	263 (97.4)
	M1	5 (1.0)	7 (2.6)
	MX	10 (2.0)	0 (0.0)
Grade (%)	NA	3 (0.6)	−
	G1	62 (12.4)	−
	G2	299 (59.7)	−
	G3	119 (23.8)	−
	G4	2 (0.4)	−
	GX	16 (3.2)	−
Fustat (%)	Alive	282 (56.3)	176 (65.2)
	Dead	219 (43.7)	94 (34.8)
HPV (%)	NA	398 (79.4)	1 (0.4)
	Negative	72 (14.4)	209 (77.4)
	Positive	31 (6.2)	60 (22.2)

*SD: standard deviation.*

The cBio Cancer Genomics (Cerami et al., 2012), Gene Expression Profiling Interactive Analysis (GEPIA) (Tang et al., 2019), and Human Protein Atlas database (HPA) (Uhlén et al., 2015) were also used to validate DNA, mRNA, and protein expression level of CMGs in HNSCC.

### Differentially Expressed Genes Analysis and Function Enrichment Analysis

By package limma (Ritchie et al., 2015), differentially expressed genes (DEGs) were screened out with the cutoff value *p* value < 0.05 and log2 (| fold change (FC)|) > 0.5. Principal component analysis (PCA) was also employed to demonstrate expression patterns of CMGs in different samples. Pathway and function enrichment analysis was performed via clusterProfiler (Yu et al., 2012). Kyoto Encyclopedia of Genes and Genomes (KEGG) (Kanehisa et al., 2017), Gene set enrichment analysis (GSEA) (Subramanian et al., 2005), and Gene Ontology (GO) enrichment analyses were employed.

### Estimation of Immune Cell Infiltration and Immune Therapy Response

The tumor purity and immune infiltration level were analyzed via ESTIMATE (Yoshihara et al., 2013), leucocyte fraction (Thorsson et al., 2018), CIBERSORT (Newman et al., 2015), MCP-counter (Becht et al., 2016), EPIC (Racle et al., 2017), quanTIseq (Finotello et al., 2019), and TIMER (Li et al., 2017). Immunophenoscore (IPS) (Charoentong et al., 2017) algorithms were used to assess immunotherapy responses.

### Mutation Analysis and Tumor Mutation Burden (TMB) Analysis

Somatic variants data of HNSCC samples were analyzed by maftools (Mayakonda et al., 2018). Then, the tumor mutation burden (TMB) of each patient (mutations per million bases) was calculated.

### Signature Identification

CMGs-based signature was constructed using 9 CMGs with a linear combination of their expression values. These inputs were weighted with the regression coefficients from the stepwise Cox regression analyses.

$$\mathrm{Risk}\mathrm{Score}=\int \sum_{n=1}^{9}\left(\mathrm{coefficient}\left(\mathrm{gene}i\right)*\mathrm{expression}\left(\mathrm{genei}\right)\right)$$

### Construction and Validation of a Prognostic Nomogram

Nomogram plotted by RMS package Before constructing the nomogram, 4 patients were excluded because of undefined pathological diagnosis. Then CMS and nomogram were tested by receiver operating characteristic (ROC) curves and calibration curves. And area under curve (AUC) values of ROC were also calculated.

### Statistical Analysis

The results were expressed as the mean ± standard deviation. Student’s *t*-test or rank-sum test was used for comparisons between groups. Categorical data were analyzed by the chi-square test or Fisher’s exact test. Correlation between two groups was determined by analysis of Pearson’s or Spearman’s correlation coefficient. Survival differences between the two groups were assessed by Kaplan–Meier method and compared using log-rank statistical methods. All statistical tests were bilateral with *p* value < 0.05 being statistically significant. R software (4.0.4) and GraphPad Prism 7 were used for data analyses.

## Results

### Genomic Features and Prognostic Value of CMGs in HNSCC

**FIGURE 1:**
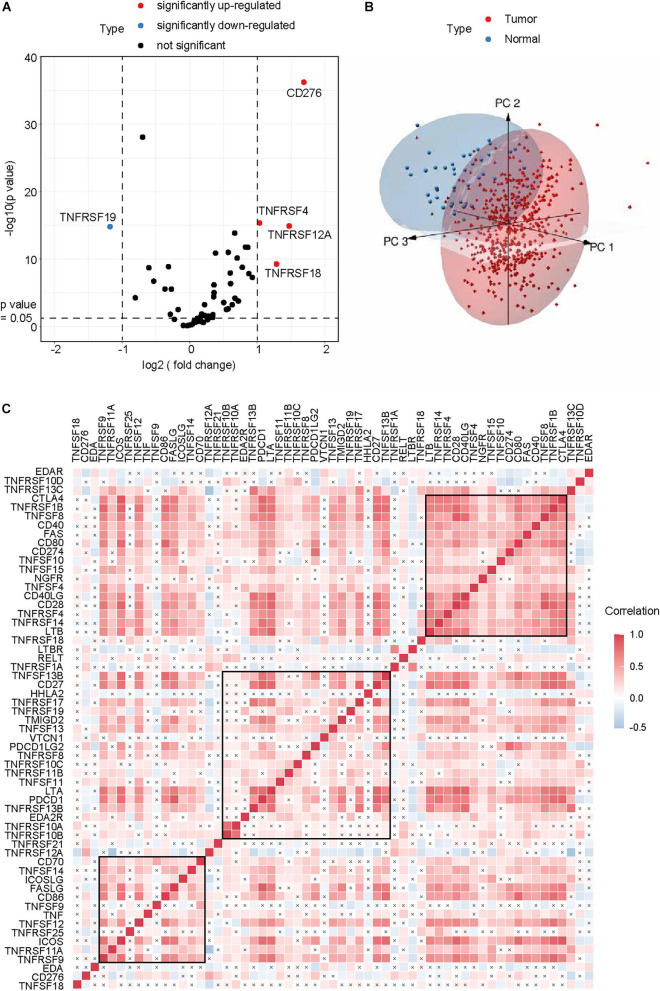
Genomic landscape of CMGs in HNSCC. **(A)** Volcano plot exhibited the different expression patterns of CMGs between HNSCC tumors and normal tissues. **(B)** PCA for the expression profiles of CMGs to distinguish HNSCC tumors from normal tissues. **(C)** Correlation analysis of CMGs in HNSCC by Spearman. The black cross represented the *p* value > 0.05.

After excluding TNFRSF6B for its low expression, a total of 59 CMGs were abstracted from The Cancer Genome Atlas (TCGA) HNSCC data, including 13 well-defined B7-CD28 family costimulatory molecules and 46 TNFRSF family costimulatory molecules (Table 2). The different expression levels of CMGs between HNSCC tumor and normal tissues were exhibited in Figure 1A and Table 2. Moreover, PCA exhibited that CMGs could obviously distinguish tumor samples from normal samples (Figure 1B). The correlation of CMGs was shown in Figure 1C. CMGs with genetic alterations rate >3% were demonstrated based on cBioPortal database (Supplementary Figure 1).

**TABLE 2:** Difference analysis and Cox analysis of costimulatory molecule genes in TCGA cohort.

Symbol	Family	Difference analysis (tumor vs. normal)		Univariate Cox analysis		
		log2 (fold change)	*p* value	HR	CI (95%)	*p* value
CD27	TNFRSF	0.57	0.001	0.776	0.687–0.876	<0.001
CD274	B7	0.544	0.001	1.012	0.9–1.137	0.847
CD276	B7	1.689	<0.001	1.206	1.007–1.446	0.042
CD28	CD28	0.179	0.008	0.595	0.44–0.804	0.001
CD40	TNFRSF	0.317	0.021	1.014	0.883–1.163	0.847
CD40LG	TNFSF	<0.001	<0.001	0.442	0.282–0.691	<0.001
CD70	TNFSF	0.928	<0.001	1.07	0.949–1.206	0.266
CD80	B7	0.382	<0.001	0.939	0.645–1.369	0.745
CD86	B7	0.605	<0.001	1.02	0.854–1.218	0.827
CTLA4	CD28	0.86	<0.001	0.725	0.606–0.868	<0.001
EDA	TNFSF	<0.001	0.002	1.488	1.091–2.028	0.012
EDA2R	TNFRSF	<0.001	<0.001	1.345	0.913–1.982	0.134
EDAR	TNFRSF	<0.001	<0.001	1.072	0.866–1.326	0.522
FAS	TNFRSF	<0.001	0.061	0.959	0.817–1.127	0.612
FASLG	TNFSF	0.222	0.013	0.758	0.594–0.966	0.025
HHLA2	B7	<0.001	0.633	1.134	0.667–1.928	0.643
ICOS	CD28	0.601	<0.001	0.696	0.566–0.855	0.001
ICOSLG	B7	0.021	0.513	0.762	0.419–1.386	0.374
LTA	TNFSF	0.222	<0.001	0.6	0.43–0.837	0.003
LTB	TNFSF	0.629	<0.001	0.814	0.718–0.923	0.001
LTBR	TNFRSF	0.355	<0.001	1.344	1.054–1.714	0.017
NGFR	TNFRSF	<0.001	0.656	1.008	0.918–1.108	0.863
PDCD1	CD28	0.274	0.018	0.764	0.638–0.914	0.003
PDCD1LG2	B7	0.876	<0.001	1.012	0.88–1.165	0.866
RELT	TNFRSF	0.667	<0.001	1.267	0.98–1.639	0.071
TMIGD2	CD28	0.092	0.177	0.653	0.468–0.911	0.012
TNF	TNFSF	0.265	0.066	0.962	0.829–1.115	0.605
TNFRSF10A	TNFRSF	0.16	0.126	1.076	0.889–1.301	0.453
TNFRSF10B	TNFRSF	0.827	<0.001	1.005	0.841–1.202	0.953
TNFRSF10C	TNFRSF	0.116	0.13	1.104	0.845–1.443	0.469
TNFRSF10D	TNFRSF	0.343	0.01	0.987	0.844–1.153	0.865
TNFRSF11A	TNFRSF	<0.001	<0.001	0.757	0.527–1.088	0.132
TNFRSF11B	TNFRSF	0.148	0.106	0.829	0.657–1.048	0.116
TNFRSF12A	TNFRSF	1.472	<0.001	1.263	1.1–1.45	0.001
TNFRSF13B	TNFRSF	0.068	0.115	0.392	0.219–0.701	0.002
TNFRSF13C	TNFRSF	0.138	0.116	0.723	0.573–0.912	0.006
TNFRSF14	TNFRSF	<0.001	0.745	0.843	0.712–0.997	0.046
TNFRSF17	TNFRSF	0.184	0.165	0.753	0.635–0.894	0.001
TNFRSF18	TNFRSF	1.285	<0.001	0.863	0.776–0.959	0.006
TNFRSF19	TNFRSF	<0.001	<0.001	0.849	0.727–0.991	0.039
TNFRSF1A	TNFRSF	0.163	0.023	1.222	0.902–1.654	0.195
TNFRSF1B	TNFRSF	0.341	0.029	0.83	0.73–0.944	0.005
TNFRSF21	TNFRSF	0.206	0.198	0.95	0.833–1.084	0.445
TNFRSF25	TNFRSF	0.779	<0.001	0.734	0.616–0.873	0.001
TNFRSF4	TNFRSF	1.019	<0.001	0.692	0.575–0.833	<0.001
TNFRSF8	TNFRSF	0.361	<0.001	0.817	0.612–1.089	0.168
TNFRSF9	TNFRSF	0.572	<0.001	0.83	0.655–1.053	0.126
TNFSF10	TNFSF	0.721	<0.001	1.02	0.91–1.145	0.731
TNFSF11	TNFSF	0.36	<0.001	0.852	0.642–1.13	0.266
TNFSF12	TNFSF	<0.001	0.009	0.885	0.737–1.062	0.188
TNFSF13	TNFSF	<0.001	<0.001	0.97	0.789–1.193	0.774
TNFSF13B	TNFSF	0.504	<0.001	0.947	0.813–1.104	0.489
TNFSF14	TNFSF	0.112	0.044	0.805	0.56–1.156	0.24
TNFSF15	TNFSF	<0.001	<0.001	0.819	0.62–1.083	0.162
TNFSF18	TNFSF	0.282	0.04	1.014	0.884–1.163	0.844
TNFSF4	TNFSF	0.704	<0.001	0.981	0.818–1.177	0.839
TNFSF8	TNFSF	0.043	0.402	0.556	0.382–0.81	0.002
TNFSF9	TNFSF	0.67	<0.001	1.004	0.876–1.149	0.958
VTCN1	B7	<0.001	<0.001	1.024	0.915–1.146	0.683

*HR: hazard ratio; CI: confidence interval.*

Then, Cox regression analysis revealed that 22 genes were highly associated with OS (*p* < 0.05, Table 2). Among them, four genes had a high hazard ratio (HR > 1) and were defined as high-risk factors, while eighteen genes had a low hazard ratio (HR < 1) and were defined as protective factors.

### Construction and Evaluation of a CMGs Based Risk Model

**FIGURE 2:**
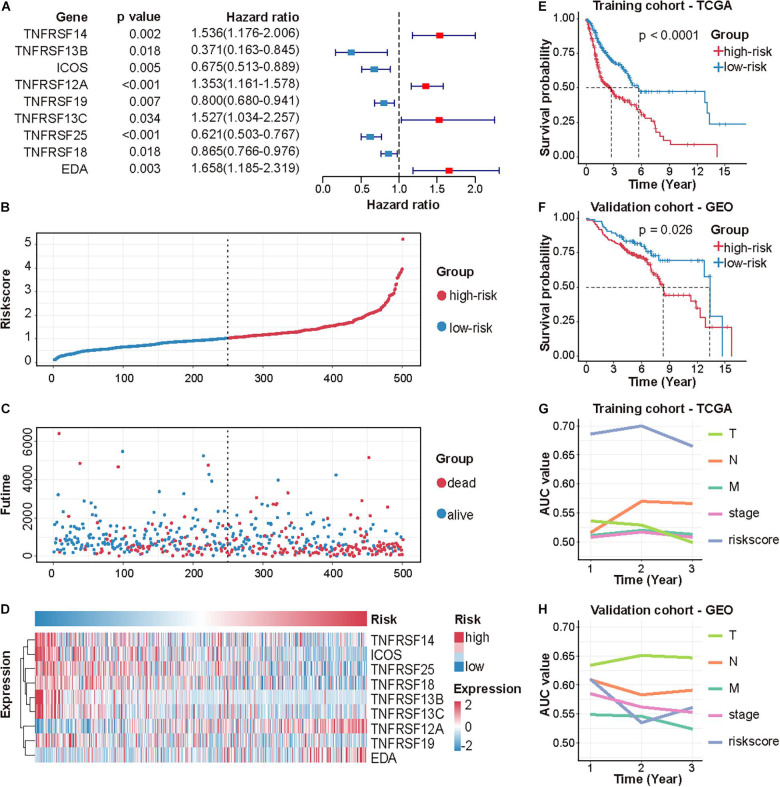
Identification and validation of CMS in HNSCC. **(A)** A forest plot of multivariate Cox regression analysis of 9 CMGs in HNSCC. The risk score distribution of each patient **(B)**, the survival time and status of each patient **(C)**, and the expression of 9 CMGs in each patient **(D)**. **(E,F)** Survival analysis of CMS in the TCGA and GEO dataset by performing the log-rank test. **(G,H)** AUC values of CMS and other clinical indices were determined in the TCGA and GEO dataset by performing ROC curve analysis.

Following stepwise Cox proportional hazards regression analysis, nine genes were selected to construct CMS (Figure 2A). Then, we established a predictive model on the basis of coefficients and expressions.

Patients were assigned to either high- or low-risk group using the median risk score as the cutoff value. The distribution of risk scores, survival status, and survival time of patients was exhibited by scatter plots (Figures 2B,C). The expressions of nine selected genes were visualized by a heat map (Figure 2D).

Differences in clinicopathologic features between the high-risk and low-risk subgroups were displayed in Table 3. Survival analysis indicated that patients in the high-risk group exhibited poorer OS (*p* < 0.0001; Figure 2E). The ROC curve for CMS and other clinical indices was shown in Figure 2G. Furthermore, the accuracy of CMS was validated in another independent GSE65858 cohort (Figures 2F,H).

**TABLE 3:** Clinical characteristics of HNSCC patients by different CMS groups.

Characteristic	Level	TCGA cohort			GEO cohort		
		High	Low	*p* value	High	Low	*p* value
Gender (%)	Female	67 (26.8)	67 (26.7)	1	29 (15.7)	18 (21.2)	0.35
	Male	183 (73.2)	184 (73.3)		156 (84.3)	67 (78.8)	
Stage (%)	I	4 (1.6)	16 (6.4)	0.017	15 (8.1)	3 (3.5)	0.304
	II	46 (18.4)	50 (19.9)		28 (15.1)	9 (10.6)	
	III	50 (20.0)	59 (23.5)		23 (12.4)	14 (16.5)	
	IV	150 (60.0)	126 (50.2)		119 (64.3)	59 (69.4)	
Grade (%)	G1	29 (11.6)	33 (13.3)	0.569	–	–	–
	G2	149 (59.6)	150 (60.5)		–	–	–
	G3	64 (25.6)	55 (22.2)		–	–	–
	G4	0 (0.0)	2 (0.8)		–	–	–
	GX	8 (3.2)	8 (3.2)		–	–	–
T (%)	T1	11 (4.4)	24 (9.6)	0.02	27 (14.6)	8 (9.4)	0.446
	T2	69 (27.6)	80 (31.9)		50 (27.0)	30 (35.3)	
	T3	66 (26.4)	70 (27.9)		40 (21.6)	18 (21.2)	
	T4	104 (41.6)	77 (30.7)		68 (36.8)	29 (34.1)	
N (%)	N0	115 (46.0)	130 (51.8)	0.552	69 (37.3)	25 (29.4)	0.583
	N1	46 (18.4)	39 (15.5)		21 (11.4)	11 (12.9)	
	N2	84 (33.6)	76 (30.3)		88 (47.6)	44 (51.8)	
	N3	4 (1.6)	3 (1.2)		7 (3.8)	5 (5.9)	
	NX	1 (0.4)	3 (1.2)				
M (%)	M0	241 (96.4)	244 (97.6)	0.734	180 (97.3)	83 (97.6)	1
	M1	3 (1.2)	2 (0.8)		5 (2.7)	2 (2.4)	
	MX	6 (2.4)	4 (1.6)				
HPV (%)	Negative	30 (83.3)	42 (62.7)	0.051	143 (77.7)	53 (62.4)	0.013
	Positive	6 (16.7)	25 (37.3)		41 (22.3)	32 (37.6)	
Fustat (%)	Alive	112 (44.8)	170 (67.7)	<0.001	114 (61.6)	62 (72.9)	0.094
	Dead	138 (55.2)	81 (32.3)		71 (38.4)	23 (27.1)	
Futime	Mean (SD)	803.57 (782.10)	1015.75 (961.09)	0.007	841.10 (429.59)	974.29 (486.73)	0.024
Age	Mean (SD)	61.43 (12.04)	60.73 (11.77)	0.509	60.03 (10.67)	60.33 (9.66)	0.827
Riskscore	Mean (SD)	1.65 (0.66)	0.69 (0.23)	<0.001	0.02 (0.01)	0.01 (0.00)	<0.001

*SD: standard deviation.*

**FIGURE 3:**
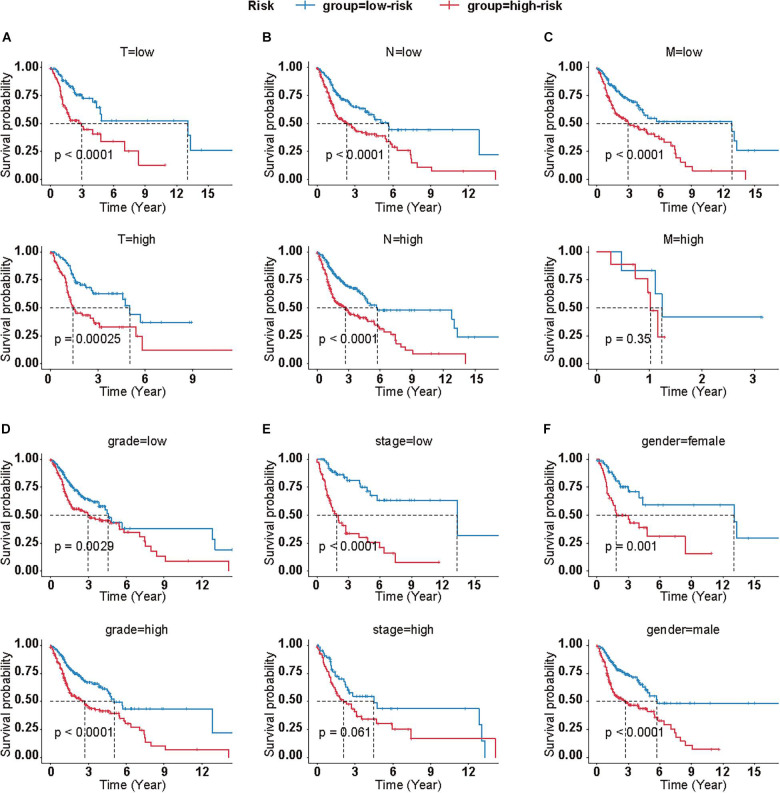
Clinical significance of CMS in different subgroups. Survival analyses of CMS in HNSCC patients with different T **(A)**, N **(B)**, M **(C)**, grade **(D)**, stage **(E)**, and gender **(F)**. (T low = T1+T2; T high = T2+T4; N low = N0+N1; N high = N2+N3+NX; M low = M0; M high = M1+MX; grade low = G1+G2; grade high = G3+G4+GX; stage low = I+II, stage high = III+IV).

Meanwhile, we calculated the correlation between clinical features and the risk score on OS, as well. Log-rank analysis manifested that higher risk scores were associated with poor prognosis in most subgroups, which was in accordance to the results in the total cohort (Figure 3).

### CMS Was an Independent Predictor for HNSCC

**FIGURE 4:**
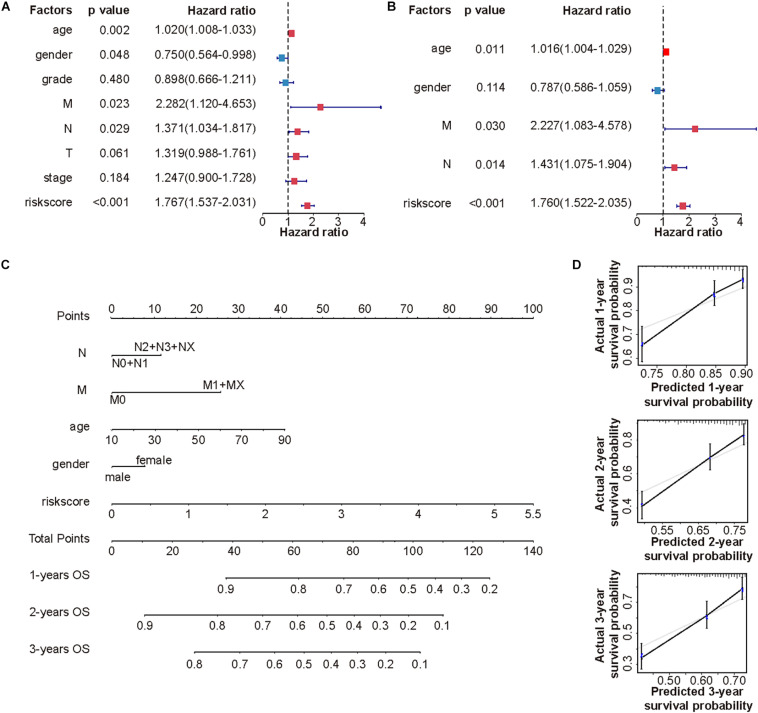
Establishment of a novel nomogram based on CMS. Forest plots exhibited univariate **(A)** and multivariate **(B)** Cox regression analysis of clinical features and risk score in the TCGA cohort. **(C)** The nomogram for predicting 1-, 2-, and 3-year OS in TCGA cohort. **(D)** Calibration curves of nomogram on consistency between predicted and observed 1-, 2-, and 3-year survival in TCGA cohort.

Subsequently, univariate analysis was used to examine the prognostic value of risk score and several clinicopathological features (Figure 4A). Consequently, risk score (*p* < 0.001), age (*p* < 0.05), gender (*p* < 0.05), N (*p* < 0.05), and M stages (*p* < 0.05) were unfavorable factors for OS. To further verify the clinical implications of CMS, multivariate Cox regression analyses were performed (Figure 4B). Collectively, these results revealed that the novel prognostic model could work as an independent prognostic factor related to the OS of HNSCC (*p* < 0.001).

### Establishment of a Novel Nomogram

To provide a more accurate estimation of survival rates for HNSCC patients, a prognostic nomogram was constructed based on statistically significant factors in univariable cox regression analysis (Figure 4C). What is more, the calibration curves of the prognostic nomogram suggested the satisfying consistency between prediction and actual 1-, 2-, and 3-year survival in the TCGA cohort (Figure 4D).

### CMS Related Biological Processes and Pathways

**FIGURE 5:**
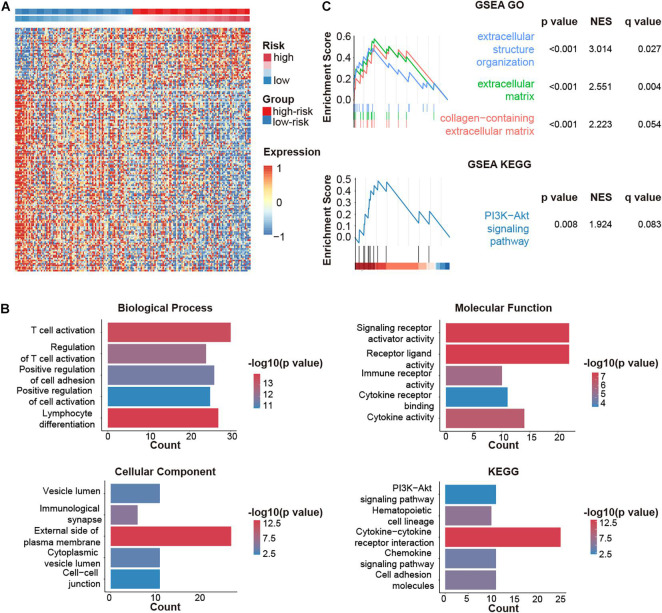
CMGs-based signature (CMS)-related biological pathways. **(A)** DEGs in different risk groups. **(B)** GO and KEGG analyses based on DEGs. **(C)** DEGs were analyzed by GSEA. NES: normalized enrichment score.

We then explored the down-stream pathways in different CMS stratification. A total of 220 DEGs were screened out. Among them, 47 genes were upregulated, while 173 genes were downregulated in the high-risk group (Figure 5A).

Gene ontology analysis revealed that DEGs were highly involved in immune-relevant responses (Figure 5B), especially immune cell activation, which was validated by GSEA (Figure 5C). Besides, KEGG and GSEA analysis jointly suggested that the PI3K-AKT pathway might be implicated during these biological processes.

### CMS Predicted Immune Infiltration and Responses of Immunotherapy

Despite the great achievements of immune checkpoint inhibitors (ICIs), only a fraction of patients could expect to benefit from such burgeoning agents. The development of predictive indices is needed to optimize patients’ benefit and avoid toxicity risks (Topalian et al., 2016). Hence, we evaluated the association of CMS and immunotherapy responses by accessing several biomarkers. Collectively, we enrolled five indices, including IPS, TMB, tumor purity, infiltration levels of different immune cells, and immune signature genes, to obtain a more comprehensive prospect.

**FIGURE 6:**
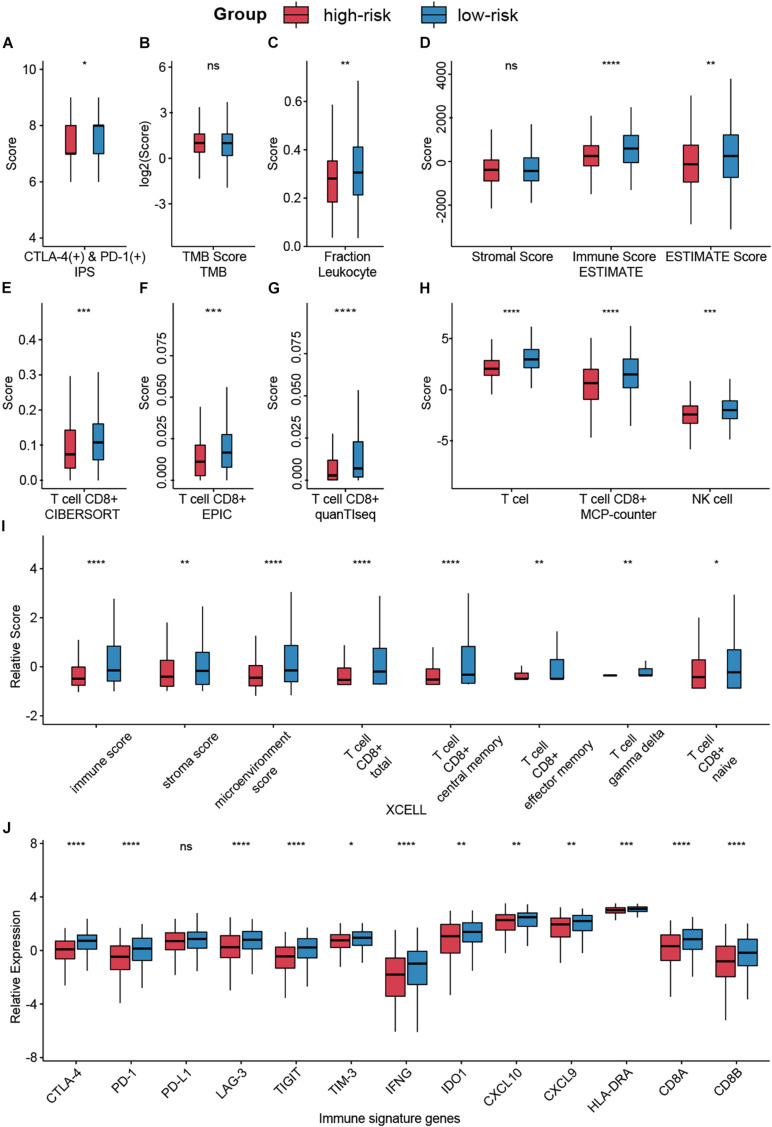
Analysis of immunotherapy responses in different risk groups. **(A)** The relationship between the risk groups and IPS. **(B)** TMB in different risk groups. **(C,D)** Leukocyte fraction and ESTIMATE score indicated that immune cells were highly enriched in the low-risk group. **(E–I)** Infiltrating immune cell in different groups was calculated by CIBERSORT, EPIC, quanTIseq, MCP-counter, and XCELL. **(J)** Expression levels of immune signature genes in different groups were exhibited by box plot. Ns: not significant, *p < 0.05, **p < 0.01, ***p < 0.001, ****p < 0.0001.

Using IPS, a machine learning-based scoring scheme to predict the responses of patients to ICIs, we found that the relative probabilities of responding to anti-PD-1/PD-L1 and anti-CTLA-4 treatment were higher in the group with low-risk scores (*p* < 0.05, Figure 6A). This indicated that patients with low-risk scores might receive more satisfactory clinical outcomes following immunotherapy. However, no significant differences between the groups were observed for TMB, which is a biomarker for immunotherapy (Chan et al., 2019; Figure 6B).

Lymphocyte infiltration, specifically CD8+ T cell and NK cell infiltration, has been documented to be associated with improved survival in various cancers (Galon et al., 2006). Based on several algorithms, such as ESTIMATE, CIBERSORT, and XCELL, infiltrating immune cells in the tumor microenvironment (TME) were calculated and exhibited in Supplementary Figure 2. Results indicated that immune cells, especially CD8+ T cell and NK cell, were highly enriched in the low-risk group (Figures 6C–I).

Besides, we compared the expression levels of immunotherapy-related genes between the high-risk score group and the low-risk score group (Figure 6J). Patients with low-risk scores had significantly elevated expression of PD-1 (*p* < 0.0001), CTLA-4 (*p* < 0.0001), and other signature genes.

### TNFRSF12A Was a Tumor Promoter in HNSCC

To investigate the significance of our risk model, we then compared the expression levels of 9 factors in CMS and found TNFRSF12A demonstrated the most significant difference between tumor and normal tissues (Table 2 and Figure 1A). Its clinical implication was further validated by GEPIA and HPA database (Supplementary Figures 3A–C). Hence, we chose TNFRSF12A for further investigation.

**FIGURE 7:**
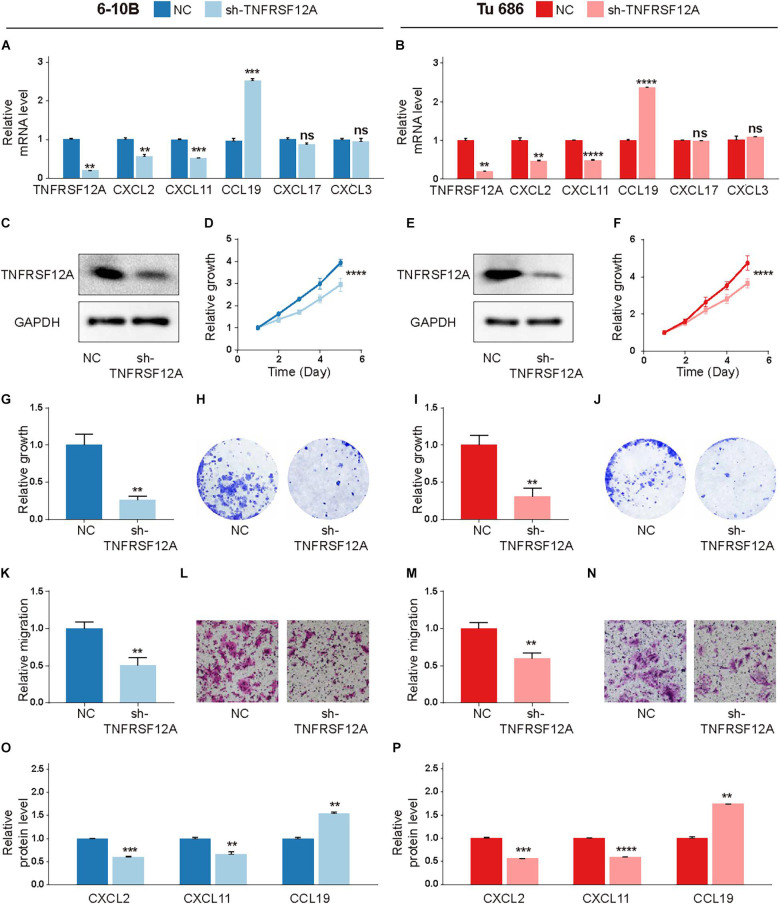
TNFRSF12A was a tumor promoter in HNSCC. **(A,B)** Expression levels of TNFRSF12A, CXCL2, CXCL11, CCL19, CXCL3, and CXCL17 were detected via real-time PCR. **(C,E)** Transfection efficacies were validated by Western Blot. **(D,F)** Knockdown of TNFRSF12A in HNSCC cell lines inhibited cell proliferation was determined by CCK-8. **(G,I)** Clone formation assays revealed the effects of TNFRSF12A down-regulation on proliferation. **(H,J)** Representative images of clone formation. **(K,M)** Tranwell assays detected migration abilities in different groups. **(L,N)** Representative images of the transwell (Scale bar = 100 μm). **(O,P)** ELISA exhibited that TNFRSF12A regulated expressions of CXCL2, CXCL11, and CCL19. *n* = 3/group for all assays; ns: not significant, **p < 0.01, ***p < 0.001, ****p < 0.0001.

Short-hairpin RNA (shRNA) was used to inhibit TNFRSF12A expression in two typical HNSCC cell lines to evaluate biological functions of TNFRSF12A. The transfection efficacies were validated by real-time PCR (Figures 7A,B) and Western Blot (Figures 7C,E). By performing CCK-8 (Figures 7D,F) and clone formation (Figures 7G–J) assays, we found that proliferation was dampened when the expression of TNFRSF12A was inhibited. Transwell assays verified that downregulation of TNFRSF12A reduced migration abilities of HNSCC cell lines in the sh-TNFRSF12A group compared to those in the negative control (NC) group (Figures 7K–N).

Moreover, in order to further explore the relationship between TNFRSF12A and TME, we investigated the relevance between the expression levels of TNFRSF12A and chemokine genes, which were deemed to cast a crucial role in shaping TME and influence clinical outcomes of immunotherapy (Nagarsheth et al., 2017). Correlation analysis revealed that 5 chemokine genes, out of a total of 43 chemokine genes, exhibited significant correlations with TNFRSF12A (Supplementary Figure 3D). Among these 5 chemokine genes, CXCL2, CXCL3, and CXCL11 showed positive correlation with TNFRSF12A (*R* > 0.25, *p* < 0.05), while CXCL17 and CCL19 showed negative correlation (*R* < −0.25, *p* < 0.05). Real-time PCR revealed that TNFRSF12A could regulate expressions of CXCL2, CXCL11, and CCL19 (Figures 7A,B). ELISA further validated that TNFRSF12A was the up-stream regulator of CXCL2, CXCL11, and CCL19 (Figures 7O,P).

## Discussion

Immune escape and T cell exhaustion are reckoned as features in the TME, closely associated with patients’ survival (Chen and Mellman, 2017). By inhibiting the immune checkpoints, immune cells could be rejuvenated to eliminate cancer cells, which becomes a potential target for cancer immunotherapy (O’Donnell et al., 2019).

In the current study, we synchronously inspected the genome landscape and prognostic value of 59 costimulatory molecules in HNSCC patients. Through TCGA RNAseq data, 22 CMGs were discovered to be related to prognosis. Most of these CMGs had been reported to be involved in various diseases, including cancer. For instance, TNFRSF12A could result in cancer, chronic autoimmune diseases, and acute ischemic stroke via the TWEAK-TNFRSF12A axis (Winkles, 2008). Overexpression of TNFSF14 could contribute to the expansion of functional T cells to prevent the growth of human papillomavirus 16-induced tumors (Kanodia et al., 2010). High expression of ICOS in leukemia was associated with poor prognosis and might contribute to tumor proliferation by assisting tumor cells in evading antitumor immune responses (Tamura et al., 2005).

Among 9 CMGs in CMS, TNFRSF12A showed the most notable difference between tumor and normal tissues. And two independent databases also verified its clinical manifestations. Thus, TNFRSF12A was chosen for subsequent experiments. By knocking down TNFRSF12A in two typical HNSCC cell lines, we found that reduced expression of TNFRSF12A significantly inhibited cellular proliferation *in vitro*. Simultaneously, downregulation of TNFRSF12A also dampened the migration ability of HNSCC cell lines, consolidating its role as a tumor promoter in HNSCC carcinogenesis. Moreover, we analyzed the association between TNFRSF12A and TME, from the perspective of chemokines. By correlation analysis, we found 5 chemokines were highly associated with TNFRSF12A in HNSCC tissues. What is more, real-time PCR and ELISA verified that TNFRSF12A could regulate CXCL2, CXCL11, and CCL19 expression, highlighting the role of TNFRSF12A in modulating TME.

Following this, the risk model based on CMGs was constructed by the TCGA data set and validated by the GEO data set. The survival analysis showed that patients with low-risk scores tended to have a higher OS rate in both validation and training cohorts. Similar results amongst HNSCC patients of different stages, grades, genders, and ages highlighted the CMS’s accuracy. Moreover, cox regression analysis confirmed the independent prognostic value of CMS, henceforth emphasizing the reliability of our risk model. To further enhance the accuracy of prognostic prediction, a novel nomogram combining clinical characteristics and CMS was constructed, which not only helped to predict the specific survival risk of specific patients but also contributed to individualized treatments for HNSCC patients. Then, pathway enrichment analyses were used to provide additional insights into the downstream pathways distinct in two groups. The GO and GSEA analyses jointly showed that DEGs were related to immune responses. And the KEGG analysis suggested that PI3K–AKT pathway, which has been reported to induce specific immune-inhibitory molecules’ expression (Pardoll, 2012), might be involved in these biological processes.

Preliminary data from trials of ICIs in the treatment of metastatic or recurrent HNSCC led to encouraging results (Ferris et al., 2016; Gillison et al., 2016; Mehra et al., 2018). Nevertheless, with the rapid augment in the utilization of ICIs, immune-related adverse events and the limited response rate for the majority of HNSCC ensued (Lyon et al., 2018; Postow et al., 2018). As a consequence of these results, biomarkers to guide patient selection for immunotherapy are attached to most priority (Bruni et al., 2020), which prompted us to investigate how our risk model would relate to immunotherapy response. Nevertheless, it is not feasible to accurately predict the response probability for ICI immunotherapy based on merely one parameter and without taking other factors into consideration (Havel et al., 2019). This is probably due to the heterogeneity of HNSCC and its TME. Thus, through integrating a series of promising indices in immunotherapy, including IPS, TMB (Samstein et al., 2019), tumor purity (Fridman et al., 2012), immune cell component (Galon et al., 2006), and immune genes (Andrews et al., 2017; Rowshanravan et al., 2018; Wolf et al., 2020), we came to the conclusion that patients with high-risk scores were inclined to acquire miserable therapeutic outcomes of ICI immunotherapy, in accordance to the results of OS.

However, there are some limitations in our study. Firstly, although an earnest endeavor was made to recruit as many HNSCC patients as possible to establish and validate this CMS model, especially considering the relatively low incidence rate of HNSCC compared to other cancers like colon cancer, this study was still a retrospective analysis. Secondly, because of the limited accessibility to acquire paired mRNA expression data from HNSCC samples before and after immunotherapy, the prediction of immunotherapy response based on CMS was estimated indirectly. The accuracy was remained restricted. Future prospective studies based on genome data could depict a more delicate landscape of the CMGs in HNSCC.

## Conclusion

This study identified expression pattern and prognostic value of CMGs in HNSCC. TNFRSF12A was found to be a tumor promoter via *in vitro* experiments. A novel scoring system based on CMGs was established to predict the clinical outcomes of HNSCC patients. It could serve as a biomarker and immunotherapy indicator for doctors to assign individualized therapeutics for patients in future clinical practices.

## Data Availability Statement

TCGA gene expression profiles and patients’ clinical data in this study are available at UCSC Xena (https://xena.ucsc.edu/). Gene mutation data could be acquired from TCGA data portal (https://portal.gdc.cancer.gov/). The patients’ IPS values are available in the Cancer Immunome Atlas (https://tcia.at/home). The immunohistochemistry results could be obtained from HPA (https://www.proteinatlas.org/). Part of genome analyses could be obtained from GEPIA (http://gepia2.cancer-pku.cn/), cBioPortal (https://www.cbioportal.org/), and Broad GDAC Firehose (http://gdac.broadinstitute.org/).

## Author Contributions

LA performed the bioinformatics analysis, drafted the manuscript, and conducted the experiments. XSo and JY collected the related references and edited the manuscript. JZ, XSu, and LH participated in the discussion. QL, HY, and DW conceived and designed the study. All authors read and approved the final manuscript.

## Conflict of Interest

The authors declare that the research was conducted in the absence of any commercial or financial relationships that could be construed as a potential conflict of interest.

## Publisher’s Note

All claims expressed in this article are solely those of the authors and do not necessarily represent those of their affiliated organizations, or those of the publisher, the editors and the reviewers. Any product that may be evaluated in this article, or claim that may be made by its manufacturer, is not guaranteed or endorsed by the publisher.

## Supplementary Material

Supplementary Figure 1 Genetic alternations of CMGs in HNSCC.

Supplementary Figure 2 Estimation of tumor-infiltrating lymphocytes in HNSCC.

Supplementary Figure 3 Clinical significance of TNFRSF12A and its correlation with chemokine genes. Clinical significance of TNFRSF12A was validated via GEPIA **(A)** and HPA **(B)**. **(C)** Representative images of immunohistochemistry of TNFRSF12A in HNSCC tissues via HPA (Scale bar = 200 μm). **(D)** Volcano plot exhibited correlation between TNFRSF12A and chemokines in HNSCC tissues by Spearman analysis.

## Abbreviations

HNSCC: head and neck squamous cell carcinoma
CMGs: costimulatory molecule genes
TCGA: The Cancer Genome Atlas
GEO: Gene Expression Omnibus
CMS: CMGs-based signature
ICIs: immune checkpoint inhibitors
TNF: tumor necrosis factor
TNFSF: TNF ligand superfamily
TNFRSF: TNF receptor superfamily
ROC: receiver operating characteristic
DEGs: differentially expressed genes
GO: Gene ontology
GSEA: gene set enrichment analysis
KEGG: Kyoto Encyclopedia of Genes and Genomes
IPS: immunophenoscore
TMB: tumor mutation burden
shRNA: short-hairpin RNA
CCK-8: cell counting kit-8
NC: negative control
OS: overall survival
HR: hazard ratio
FC: fold change
PCA: principal component analysis
TME: tumor microenvironment
ELISA: enzyme-linked immunosorbent assay
AUC: area under curve
NES: normalized enrichment score.
