# Supplementary figures and images for: Identification of a Costimulatory Molecule Gene Signature to Predict Survival and Immunotherapy Response in Head and Neck Squamous Cell Carcinoma
Source: Front Cell Dev Biol. 2021 Aug 9;9:695533. doi: 10.3389/fcell.2021.695533 (PMC8381651; doi:10.3389/fcell.2021.695533)

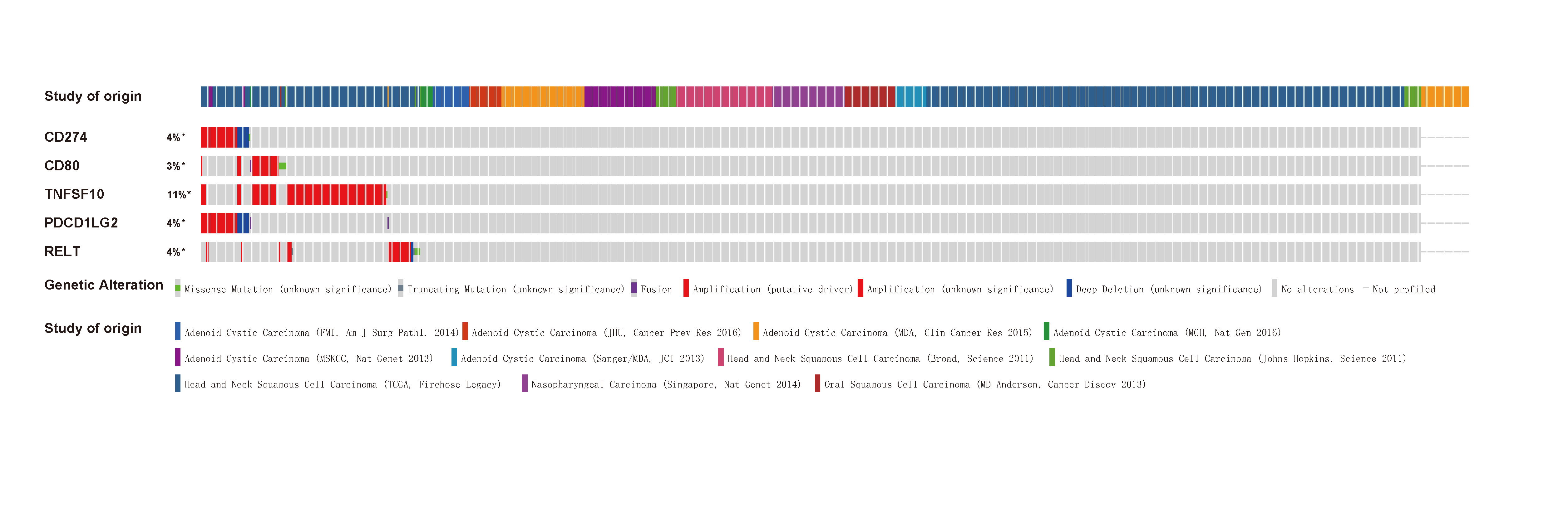

Supplement: Supplementary Figure 1 — Genetic alternations of CMGs in HNSCC. [file Image_1.tiff]

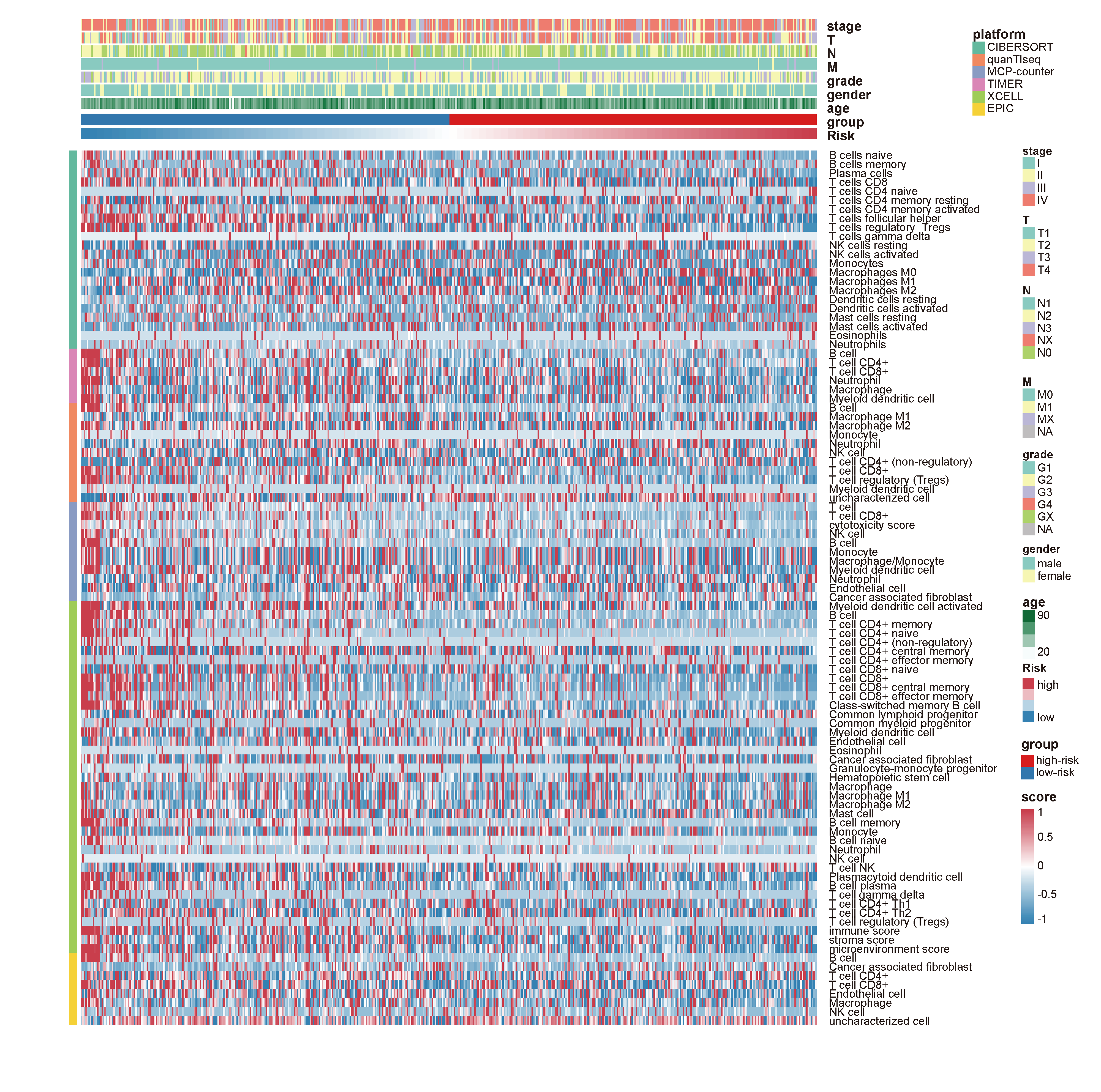

Supplement: Supplementary Figure 2 — Estimation of tumor-infiltrating lymphocytes in HNSCC. [file Image_2.tiff]

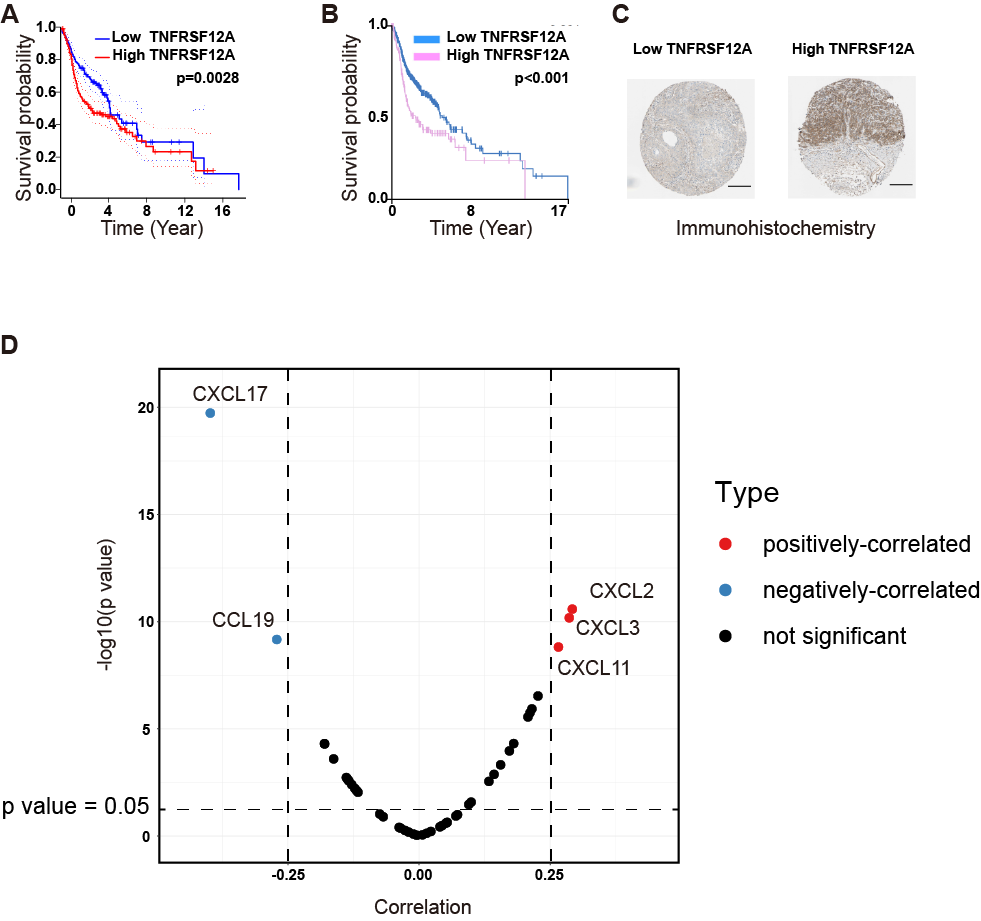

Supplement: Supplementary Figure 3 — Clinical significance of TNFRSF12A and its correlation with chemokine genes. Clinical significance of TNFRSF12A was validated via GEPIA (A) and HPA (B). (C) Representative images of immunohistochemistry of TNFRSF12A in HNSCC tissues via HPA (Scale bar = 200 μm). (D) Volcano plot exhibited correlation between TNFRSF12A and chemokines in HNSCC tissues by Spearman analysis. [file Image_3.tiff]
